# Retreatment of a Mandibular Second Premolar with Three Roots: A Case Report

**Published:** 2014-03-08

**Authors:** Eshagh Ali Saberi, Hossein Rasooli, Zeinab Movassagh

**Affiliations:** a * Department of Endodontics, Dental School, Zahedan University of Medical Science, Zahedan, Iran; *; b * Department of Endodontics, Dental School, Ilam University of Medical Science, Ilam, Iran*

**Keywords:** Anatomic Variation, Endodontic Retreatment, Three Rooted Premolar

## Abstract

Mandibular premolars have earned a reputation for having aberrant anatomy. The occurrence of three canals with three separate foramina in mandibular premolars is very rare. If predictable treatment of a three rooted mandibular premolar is planned, precise knowledge of clinical and radiographic anatomy is absolutely necessary. These teeth may also require special shaping and obturating techniques. This article reports and discusses the treatment recommendations for an unusual occurrence of three canals with three separate foramina in a second mandibular premolar.

## Introduction

Consistent and successful endodontic treatment requires an understanding of root canal anatomy and morphology. Ingle has reported that the most significant cause for endodontic failures was incomplete canal instrumentation, followed by incorrect canal obturation [1]. Slowey has indicated that because of the probable variations in canal anatomy, the mandibular premolars are the most difficult teeth to treat endodontically [2]. Variation in root canal morphology is considered as the most likely reason for the high frequency of endodontic flare-ups and failures [2, 3]. Quite a few studies were published in endodontic literature regarding the common reasons for endodontic failures [4, 5]. Hoen and Pink found a 42% incidence of missed roots or canals in the teeth that needed retreatment in their investigations. They concluded that the clinical application of a thorough knowledge of canal anatomy and meticulous attention to treatment details are essential to minimize the failure rate and the need for subsequent endodontic retreatment [6].

Between 1970s and 1980s and in a series of studies on extracted teeth, Vertucci determined canal numbers and configurations by percentages for each of the teeth [7]. Vertucci’s data may not be exactly representative of different locations and ethnic groups, but it is a good starting point for understanding root canal anatomy.

The mandibular second premolar is typically described in textbooks as a single rooted tooth with a single root canal system [3, 8]. Over the past decades, many studies have looked at the root canal morphology of mandibular premolars and have reported multiple canals in fairly high percentage of these teeth [9, 10]. Root morphology and canal configuration of the mandibular second premolars can be extremely complex and highly variable [8, 11-16]. The factors that can contribute to observed differences in the various anatomic studies include ethnicity [17], age [18], gender [19] and study design (*in vitro* versus *in vivo*). Vertucci and Zillich *et al*. reported the occurrence of three canals in mandibular second premolars to be 0% and 0.4%, respectively [20, 21]. This case report represents the challenges in retreatment of a second mandibular premolar with three canals and three separate foramina.

## Case Report

**Figure 1:**
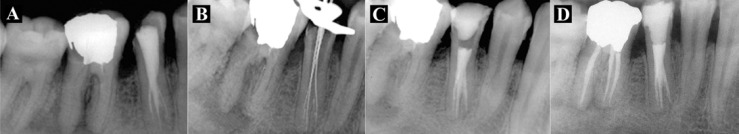
*A)* Preoperative diagnostic radiograph; *B)* Working length determination radiograph; *C)* Final radiograph; *D)* Six-month follow-up; the tooth #46 was also retreated within this time period

A 25-year-old female was referred to the department of endodontics, Zahedan Dental University. The patient’s chief complaint was a fractured amalgam restoration on the mandibular right second premolar, *i.e.* tooth #45 (Figure 1A). The medical history was noncontributory. Clinical examination revealed a defective amalgam filling with missing coronal seal in the right mandibular second premolar and first molar (#45 and #46). The tooth #45 was mildly tender to percussion. Preoperative radiograph showed incomplete and poor obturation of the canal in the premolar tooth. A diagnosis of chronic apical periodontitis was made and endodontic retreatment was planned for tooth #45. The patient was encouraged to seek for endodontic retreatment of the adjacent molar, as well.

After administration of the local anesthesia using 2% lidocaine with 1:80000 epinephrine (Daroupakhsh, Tehran, Iran), the tooth #45 was accessed under rubber dam isolation. Sizes 3 to 1 of Gates Glidden drills (Dentsply, Maillefer, Ballaigues, USA) were used in a crown-down fashion to enlarge the orifices with a brushing motion. The existing root filling was removed with solvent, hand and rotary files. After determination of the working length with an apex locator device (iPex, NSK, Tochigi, Japan) and its radiographic confirmation (Figure 1B), the canals were cleaned and shaped with ProTaper files (Dentsply, Maillefer, Ballaigues, USA) supplemented with alternate 2.5% sodium hypochlorite irrigation.

Patency was achieved in all the canals and was maintained with #10 K-file (Dentsply, Maillefer, USA). After drying the canals with paper points, the master gutta-percha points were fit within the canals and confirmation radiography was taken. Canals were obturated with cold lateral compaction of gutta-percha and AH-26 sealer (Dentsply, Tulsa Dental, Tulsa, OK, USA) (Figure 1C). The access cavity was filled with amalgam. The 6-month follow-up radiography revealed healing of the periapical radiolucent lesion around tooth #45 (Figure 1D).

## Discussion

Successful and predictable endodontic treatment requires knowledge of biology, physiology and root canal anatomy. Accurate straight and angled preoperative radiographs using parallel technique, are essential in providing clues such as the number of existing roots [22].

In mandibular premolars with three canals, the cervical half of the root is generally wider than usual, with little or no taper [13-16]. All root canals may not be evident radiographically or may look unusual. Root canal pathway may disappear halfway through the roots. Careful interpretation of the periodontal ligament space may suggest the presence of an extra root or canal. Mesial- and distal-angled radiographic views will often reveal the presence of a bi/trifurcation of the root canal [13-16, 23]. The use of magnification and fiber optic illumination, offers a tremendous advantage in locating and treating these “extra” canals. Also the dental operating microscope (DOM), has been found to be particularly helpful [24]. Cone-beam Computed Tomography (CBCT) technology provides some information concerning extra canals, apical deltas, canal morphology, accurate measurement potential in all aspects of root canal system, and a three-dimensional image of root canal(s) anatomy [25].

Optimum opening of the access cavity is absolutely necessary. During the initial placement of scouting files (hand K-files #6, 8, or 10) in the main canal, an obstruction may be encountered halfway and the file may deflect to the buccal or lingual before it travels any further. This may indicate a canal division. Thereafter, it is important to develop a sense of tactile feel and direction with appropriately precurved scouting files to detect the bi/trifurcation when working with the DOM. One can also see the hypochlorite bubbling in the extra canal, marking its presence (champaign bubbling test). Sometimes, dyes or transillumination may be helpful in locating additional canals [26]. Once it has been established that there are multiple canals, it is important to obtain straight line access to all of them. This may be achieved by Gates-Glidden drills set on a low speed handpiece in a crown-down fashion. The working length may be determined using radiographs and electronic apex locators. Despite the existence of complicated dental anatomy, shaping outcomes with nickel-titanium instruments are mostly predictable [25]. Cautious use of rotary or hand nickel-titanium files prepares the canals to a predetermined shape [27]. It also requires proper instruments and the knowledge to use these instruments effectively.

## Conclusion

High level of success in endodontic treatment requires an understanding of root canal anatomy and morphology. The clinician should have a thorough knowledge of root canal anatomy to identify the presence of anatomic variations and extra canals.

## References

[1] Ingle JI (1961). A standardized endodontic technique utilizing newly designed instruments and filling materials. Oral Surg Oral Med Oral Pathol.

[2] Slowey RR (1979). Root canal anatomy. Road map to successful endodontics. Dent Clin North Am.

[3] Ingle J, Bakland L (2002). Endodontics.

[4] Swartz DB, Skidmore AE, Griffin JA Jr (1983). Twenty years of endodontic success and failure. J Endod.

[5] Vire DE (1991). Failure of endodontically treated teeth: classification and evaluation. J Endod.

[6] Hoen MM, Pink FE (2002). Contemporary endodontic retreatments: an analysis based on clinical treatment findings. J Endod.

[7] Vertucci FJ (1984). Root canal anatomy of the human permanent teeth. Oral Surg Oral Med Oral Pathol.

[8] Ash M, Nelson S (2003). Wheeler’s dental anatomy, physiology and occlusion.

[9] Amos ER (1955). Incidence of bifurcated root canals in mandibular bicuspids. J Am Dent Assoc.

[10] Baisden MK, Kulild JC, Weller RN (1992). Root canal configuration of the mandibular first premolar. J Endod.

[11] Brown P, Herbranson E (2005). Dental anatomy &3D atlas version3.0.

[12] Cleghorn BM, Christie WH, Dong CC (2006). Root and root canal morphology of the human permanent maxillary first molar: a literature review. J Endod.

[13] Asgary S (2006). Endodontic therapy in a three canal mandibular second premolar. Iran Endod J.

[14] Borna Z, Rahimi S, Shahi S, Zand V (2011). Mandibular second premolars with three root canals: a review and 3 case reports. Iran Endod J.

[15] Kakkar P, Singh A (2012). Mandibular first premolar with three roots: a case report. Iran Endod J.

[16] Mokhtari H, Niknami M, Zand V (2013). Managing a mandibular second premolar with three-canal and taurodontism: a case report. Iran Endod J.

[17] Trope M, Elfenbein L, Tronstad L (1986). Mandibular premolars with more than one root canal in different race groups. J Endod.

[18] Pineda F (1973). Roentgenographic investigation of the mesiobuccal root of the maxillary first molar. Oral Surg Oral Med Oral Pathol.

[19] Sert S, Bayirli GS (2004). Evaluation of the root canal configurations of the mandibular and maxillary permanent teeth by gender in the Turkish population. J Endod.

[20] Vertucci FJ (1978). Root canal morphology of mandibular premolars. J Am Dent Assoc.

[21] Zillich R, Dowson J (1973). Root canal morphology of mandibular first and second premolars. Oral Surg Oral Med Oral Pathol.

[22] Silha RE (1968). Paralleling long cone techic. Dent Radiogr Photogr.

[23] Shalavi S, Mohammadi Z, Abdolrazzaghi M (2012). Root canal treatment of maxillary and mandibular three-rooted premolars: a case report. Iran Endod J.

[24] Carr GB (1992). Microscopes in endodontics. J Calif Dent Assoc.

[25] Peters OA (2004). Current challenges and concepts in the preparation of root canal systems: a review. J Endod.

[26] Nallapati S, Glassman G (2004). Use of ophthalmic dye in root canal location. Endodontic practice.

[27] Venturi M, Breschi L (2004). Evaluation of apical filling after warm vertical gutta-percha compaction using different procedures. J Endod.

